# Affordable Indocyanine Green Imaging: A Handmade Low-Cost Camera for Lymphatic Surgery Application

**DOI:** 10.1093/asjof/ojag042

**Published:** 2026-02-27

**Authors:** Sina Heymans, Maximilian Mahrhofer, Magdalena Lewicki, Bernadette Muth, Sven Lamprecht, Thomas Schoeller, Laurenz Weitgasser

## Abstract

**Background:**

Indocyanine green (ICG) has become an essential tool in lymphatic and vascular surgery because of its ability to provide real-time, near-infrared fluorescence imaging of both lymphatic structures and blood vessels. This technology enhances intraoperative decision making by allowing the surgeon to evaluate tissue perfusion, visualize lymphatic and blood channels, and confirm the patency of vascular anastomosis. Owing to these advantages, its use has become increasingly popular in lymphaticovenous anastomosis procedures and flap-based reconstructions. However, commercially available ICG imaging systems are associated with high costs, which limits accessibility to this technology.

**Objectives:**

To address this limitation, the authors of this study developed a cost-effective camera system for real-time ICG angiography.

**Methods:**

In this paper, the authors describe the technical components and assembly process of their camera system. They also share their initial intraoperative experiences with this device in a series of lymphatic surgeries.

**Results:**

Early results demonstrate that the camera provides clear imaging of lymphatic channels and blood vessels at a fraction of the cost of standard commercial systems. Its use is reliable and beneficial for intraoperative visualization.

**Conclusions:**

The budget-friendly, handmade ICG camera may deliver reliable intraoperative imaging of lymphatic and vascular structures. This approach may broaden the access to ICG technology in resource-limited environments.

**Level of Evidence: 5 (Therapeutic):**

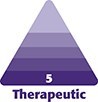

Indocyanine green (ICG) is a water-soluble fluorescent dye first developed by Kodak Research Laboratories (Eastman Kodak Company, Rochester) in the mid-20th century.^1,2^ Initially, it was introduced for assessing hepatic function and measuring cardiac output.^1^ Since then, it has been used in a wide range of medical applications because of its nontoxicity, its low rate of complications, its short half-life of ∼3 to 4 min, and its rapid hepatic clearance.

These characteristics, combined with favorable optical properties, have led to its widespread adoption in diagnostics and surgical procedures since its introduction in 1955.^2,3^

ICG fluorescence enables visualization of anatomical structures beneath the skin or within tissues, thereby improving diagnostic accuracy and surgical precision.

In plastic and reconstructive surgery, ICG has proven valuable for evaluating tissue viability.^4^ By binding to blood lipoproteins and remaining largely intravascular, ICG allows real-time evaluation of blood flow and tissue perfusion, helping surgeons make safer intraoperative decisions.^3^ This is particularly important in flap-based reconstructive surgeries, lymphovenous bypass procedures, and tumor resections. Apart from the mentioned applications, its use expanded into oncology, where it aids in sentinel lymph node mapping and tumor margin identification.^3^ Further, it is successfully used in cholangiography, neuro- and ophthalmic surgery.^2^

Commercially available operating systems for ICG imaging are often costly and can be financially challenging for medical institutions. For instance, the SPY Elite system (Stryker Corporation, MI), a widely used fluorescence imaging platform, costs up to ∼275,000 USD, whereas the Hamamatsu PDE-Probe (Hamamatsu Photonics K.K., Shizuoka Prefecture, Japan), another popular model, is priced ∼76,700 USD.^5^

These high costs limit the widespread adoption of this technology in resource-constrained environments.

In response, we developed an affordable ICG camera system that has been successfully utilized in various lymphovenous bypass surgeries. The device enabled clear visualization of lymphatic vessels and confirmation of anastomotic patency.

## METHODS

Our ICG camera system includes 3 cost-sensitive components, which were purchased from IRreCams.de for the total cost of 805 euro.

### Device Components

#### Infrared Camera With Integrated Infrared Filter

The infrared camera Olympus OMD E-M10 II (Olympus Corporation, Tokyo, Japan) detects near-infrared (NIR) light, which is emitted by ICG when it is exposed to a specific wavelength of light. An integrated 800 nm long-pass infrared filter selectively filters NIR light over 800 nm, while blocking visible light, thereby isolating the fluorescence emitted by the ICG.^3^

#### Lens

To ensure optimal focus and image clarity, a standard lens Panasonic Lumix G 25 mm F1.7 ASPH (Panasonic Corporation, Osaka, Japan) was attached to the camera. The camera lens was chosen based on its favorable cost-to-performance ratio. It features a wide maximum aperture (*f*/1.7), allowing sufficient light capture under low-light conditions, and includes autofocus functionality, which simplifies intraoperative handling.

#### Red Light Source

**Figure 1.**
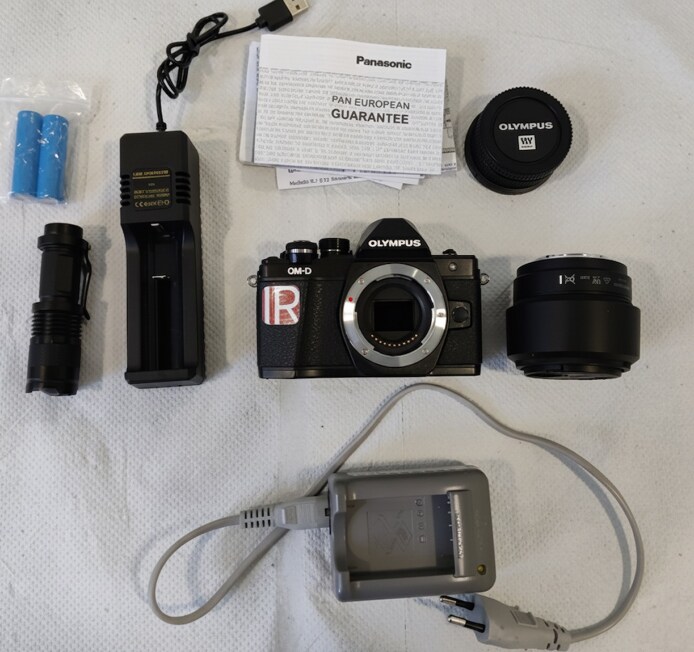
Components of our indocyanine green camera system. On the left is the red-light source (black housing). In the center is the modified Olympus OMD E-M10 II infrared camera with an integrated 800 nm long-pass filter. To the right is the Panasonic Lumix G 25 mm F1.7 ASPH lens. Further shown are accessories (batteries, charger, and documentation).

Red light can activate ICG at wavelengths as low as 600 nm. In our setup, the red-light source emits at 670 nm, effectively exciting the ICG and producing a NIR fluorescence signal, which spans ∼750 to 950 nm, depending on the excitation wavelength.^3^ The resulting fluorescence is captured by our camera, and the integrated 800 nm long-pass filter ensures that only fluorescence above 800 nm is detected, minimizing background interference from shorter wavelengths. This red-light source was selected primarily for cost-effectiveness and availability, because red-light sources at this wavelength are widely produced and readily accessible. Importantly, we note that alternative light sources with similar excitation characteristics—regardless of cost—would be expected to perform comparably (Figure 1).

### Assembly and Use of the Handheld Camera

**Figure 2.**
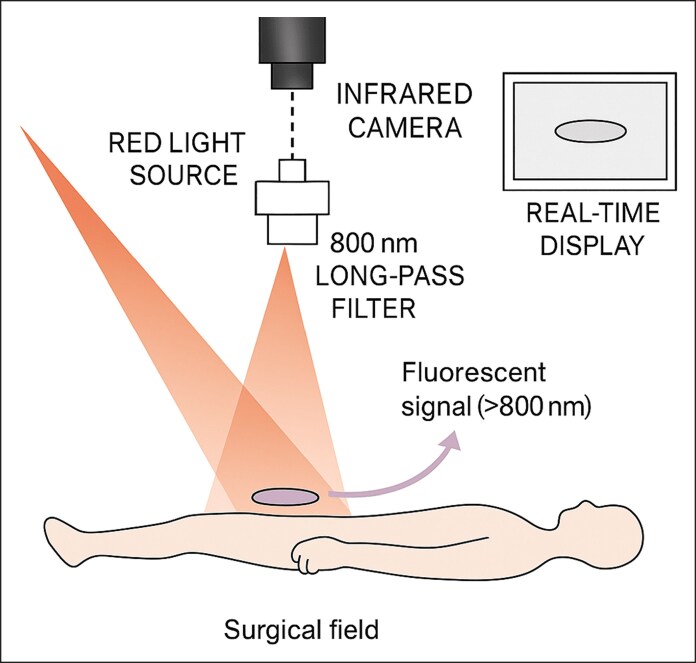
Schematic of our indocyanine green (ICG) imaging system. A red-light source excites the ICG in the surgical field, producing a fluorescent signal, which is captured with the infrared camera and the integrated 800 nm long-pass filter. The image is displayed on the monitor.

The assembly and use of our handheld camera are straightforward. The lens is attached to the camera, which is equipped with an infrared filter. The red-light source is positioned over the surgical field, ensuring the excitation of the ICG. The camera monitor displays real-time images during the procedure (Figure 2).

### Patient Selection

Patients were selected consecutively from individuals undergoing lymphatic surgery or lymphedema evaluation at our institution in whom intraoperative or diagnostic ICG imaging was clinically indicated. No additional inclusion or exclusion criteria were applied beyond standard surgical eligibility.

### Imaging Process

**Figure 3.**
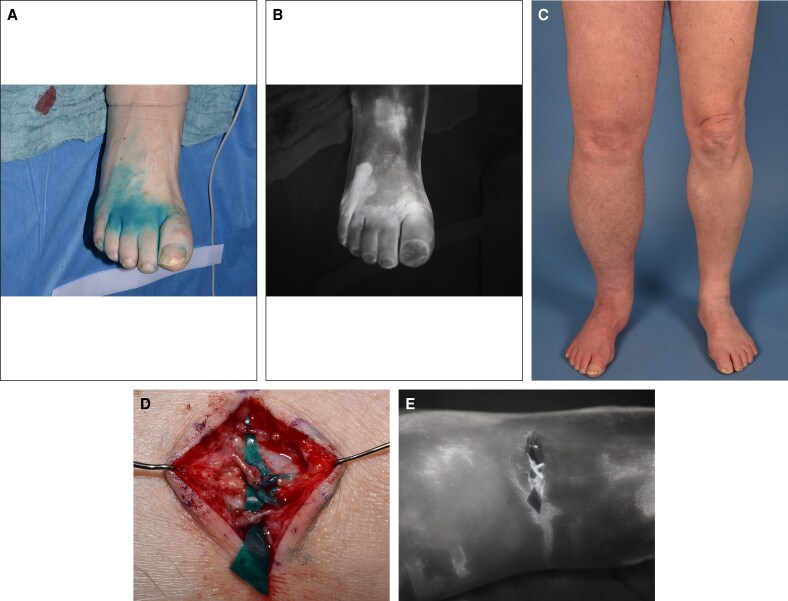
(A) The clinical view of a 76-year-old male patient's right foot following subcutaneous indocyanine green (ICG) injection into the interdigital web spaces, with (B) the corresponding lymphographic image. (C) The preoperative clinical photograph of our patient, who suffers from right lower extremity lymphedema. (D) A clinical photograph of the lymphovenous anastomosis performed intraoperatively. (E) The intraoperative lymphographic image confirms the patency of the anastomosis with clear ICG downstream flow.

Intraoperatively, up to 1 mL ICG (VERDYE 5 mg/mL; Diagnostic Green, Aschheim, Germany) is diluted with 10 mL Aqua ad iniectabilia (B. Braun, Melsungen, Germany) is injected subcutaneously at a dose of 0.1 to 0.3 mg/kg body weight into the web spaces of the upper or lower extremities, depending on the manifestation of the lymphedema.^6^ Then, massaging and lymph draining of the area, to help with the migration of the ICG downstream, are performed. In awake patients, active movement and range of motion exercises, such as short walks, can facilitate distribution of ICG. Within minutes of the injection, the dye highlights lymphatic channels and blood vessels. After about 10 min the fluorescence emitted by the ICG is captured by our camera system and displayed as a real-time image. If necessary, another 1 mL dye bolus can be administered after about 15 min.^3^ The camera system provides a clear visualization of lymphatic pathways, helping the surgeon to identify lymphatic vessels necessary for lymphatic bypass surgery. Furthermore, it enables intraoperative verification of the patency of the lymphovenous anastomosis and lymphatic uptake as well as flow (Figure 3 and Video). A short video is included demonstrating the setup and clinical use of our camera system, highlighting real-time ICG tracking and visualization of lymphatic structures.

### Diagnostic Application

**Figure 4.**
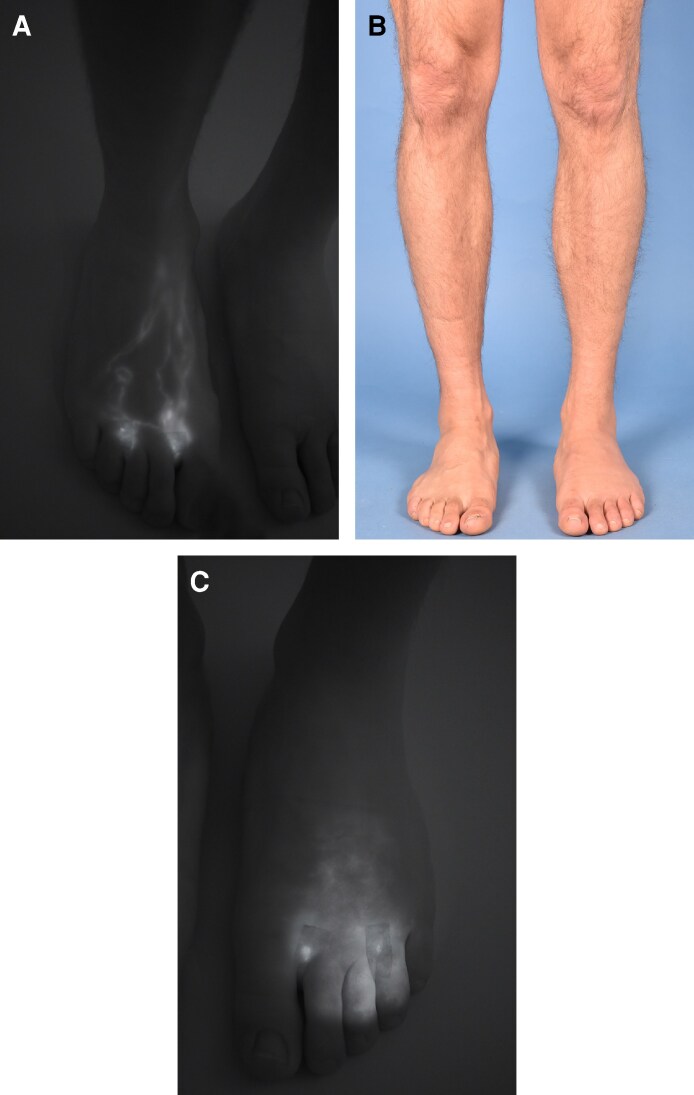
The image shows the clinical appearance of (B) the lower limbs and (A,C) the corresponding lymphographic images following interdigital injection. The patient is a 43-year-old male with lymphedema of the left foot, which is visible as diffuse dermal backflow in (C) the lymphographic image on the right. In contrast, (A) the right foot shows a healthy linear lymphatic pattern.

Beyond its intraoperative use, ICG has proven to be valuable in preoperative diagnostics. ICG lymphography helps assess and stage lymphedema. The fluorescent dye is again administered subcutaneously in the interdigital spaces or around the affected area. A real-time visualization of superficial lymphatics, with an imaging depth of 1 to 2 cm, and its distribution pattern is displayed.^1^ Nonleaking, patent lymphatic vessels show as linear patterns, dilated lymphatics are depicted as a splash, minor lymphatic extravasation is expressed as stardust, and extensive extravasation is visualized as a diffuse dermal backflow pattern.^1^ Based on those dermal backflow patterns, a qualitative staging system was introduced, which helps determine the severity of the lymphedema.^7,8^ Consequently, the adequate therapeutic steps can be addressed (Figure 4).

### Ethics Statement

The study adhered to the Declaration of Helsinki and the Guidelines for Good Scientific Practice. Measures were taken to ensure the safety of patient data and protect privacy. Written consent for the publication of the patient's pictures was obtained.

## RESULTS

In all cases, lymphatic channels and vascular structures identified using the handmade ICG camera corresponded with expected anatomical findings. The system enabled reliable identification of lymphatic vessels and confirmation of anastomotic patency in real time. Image quality was sufficient to guide surgical decision making in all procedures performed. No adverse events related to ICG administration or device use were observed.

## DISCUSSION

Do-it-yourself (DIY) ICG imaging devices have emerged in recent years and have been applied across various surgical fields.^9-12^ The device components can be easily ordered online, and assembly is straightforward. Although there might be variations in costs, size, and number of parts, the key components of self-made ICG camera devices include an NIR-sensitive camera, a monitor, a lens, an excitation light source, and a specific long-pass filter. Once assembled, these cameras offer a wide range of applications. For example, Sandor et al used their handmade device for a sentinel lymph node removal in breast surgery, to identify liver tumor margins, to evaluate perfused bowel in intestinal necrosis, to display the parathyroid glands during thyroid surgery, and to check kidney perfusion before organ transplantation.^12^ Kwon et al utilized an NIR camera for the intraoperative visualization of the vasa nervorum of the facial nerve in deep facial tissue dissection. Thus, finding a simple and efficient method to prevent facial nerve injury and resulting palsy.^13^ A study by Jørgensen et al demonstrated that ICG lymphangiography is a more accurate predictor of lymphedema severity and tissue changes following breast cancer than traditional clinical assessment, suggesting that ICG staging better reflects underlying pathophysiology and tissue remodeling.^14^ Incorporating ICG staging into routine evaluation may therefore enhance treatment planning and monitoring of disease progression.

We have used our self-made ICG camera primarily for lymphatic surgeries and lymphedema diagnostics, but we believe such a device can be useful in almost any surgical institution. Our DIY camera device has demonstrated sufficient intraoperative resolution and real-time imaging capability to support complex procedures such as lymphovenous anastomosis. The use of this device appears to reduce operative time because lymphatic vessels are identified more quickly. Moreover, surgical outcomes may improve because the patency of the anastomosis can be confirmed intraoperatively.

Regarding lymphedema diagnostics, an ICG lymphography can be easily and safely performed in an outpatient clinic, providing instant information about the severity of the lymphedema and its stage. Therefore, it reduces the need to expose the patient to more invasive diagnostic procedures such as lymphoscintigraphy. Yoon et al compared ICG lymphography with lymphoscintigraphy in secondary upper limb lymphedema and found that ICG Arm Dermal Backflow (ADB) staging strongly correlates with lymphoscintigraphy severity. The authors concluded that ADB staging is a reliable complementary tool for evaluating lymphedema severity.^15^ We also believe that postoperative ICG lymphography can be useful for evaluating improved lymphatic drainage following surgery. Overall, our ICG camera device has proven suitable for pre-, intra-, and postoperative evaluation of lymphedema patients.

### Advantages and Limitations of Do-it-Yourself ICG Systems

The primary advantage of DIY ICG cameras lies in their cost-effectiveness. The total cost of our handmade ICG camera system was ∼805 euro. In comparison, commercially available systems, such as the SPY Elite system, cost up to ∼275,000 USD, whereas the Hamamatsu PDE-Probe is priced at ∼76,700 USD, representing a cost reduction of >99% compared with high-end commercial platforms.^5^ This significant reduction in equipment cost makes this technology accessible even in underserved regions. Torgbenu et al developed practice points for healthcare workers in low- and middle-income countries to optimize lymphedema management, emphasizing skin care, exercise, lymphatic massage, and compression therapy. Surgical procedures, such as lymphaticovenular anastomosis, were excluded from their recommendations because of limited surgical expertise, inadequate facilities, and high costs.^17^ Low-cost innovations like our system may represent an important step toward establishing the infrastructure necessary for advanced surgical interventions. The financial savings achieved through DIY camera systems can be redirected toward patient education, preventive care, and expanding access to surgical treatments. Head et al compared the economic impact of complete decongestive therapy (CDT) and lymphovenous bypass in treating upper limb lymphedema and found that lymphovenous bypass can be economically justified, as the additional surgical costs may be offset by the savings from reducing or eliminating the need for ongoing CDT.^16^Furthermore, DIY systems could play a pivotal role in research by enabling more widespread data collection and analysis on different disorders, which require surgical intervention. Moreover, many DIY camera devices are lightweight and portable, offering more flexibility regarding their setup. The use even in space-limited operating rooms, field settings, or mobile clinics would be possible. Despite these advantages, DIY ICG imaging systems have limitations. Variability in camera design and the lack of standardized assembly or calibration protocols may lead to inconsistent image quality. The development of standardized protocols is essential to ensure reproducible, high-quality imaging and to encourage broader adoption. Additionally, DIY systems typically lack regulatory approval, raising concerns about clinical reliability and legal compliance. Operation and assembly require basic technical knowledge, and clinicians must be properly trained to interpret ICG images, especially when image quality varies.

## CONCLUSIONS

Our cost-effective, handmade camera system may provide reliable, real-time ICG angiography imaging of high quality during lymphatic surgeries. Its low acquisition cost and reliable intraoperative performance can potentially make it a practical option for expanding access to ICG-guided procedures in resource-limited environments. Confirmation and standardization will need to be confirmed in more extensive studies.
